# Do Oxytetracycline and Ciprofloxacin Affect Growth Phenotype, Leaf Photosynthetic Enzyme Activity, Nitrogen Metabolism, and Endogenous Hormone Homeostasis in Maize Seedlings?

**DOI:** 10.3390/plants14193021

**Published:** 2025-09-30

**Authors:** Mingquan Wang, Yong Wang, Guoliang Li, Guanghui Hu, Lixin Fu, Shaoxin Hu, Jianfei Yang, Zhiguo Wang

**Affiliations:** 1 Maize Research Institute, Heilongjiang Academy of Agricultural Sciences, Harbin 150086, China; ymswmq@haas.cn (M.W.); ziranxing1@haas.cn (G.L.); guanghuihumaize_h@haas.cn (G.H.); fulixin@haas.cn (L.F.); hushaoxin@haas.cn (S.H.); kingguo@haas.cn (Z.W.); 2 Key Laboratory of Germplasm Enhancement, Physiology and Ecology of Food Crops in Cold Region, Ministry of Education, Northeast Agricultural University, Harbin 150030, China; wyong5264@163.com

**Keywords:** maize, oxytetracycline, ciprofloxacin, seedling growth

## Abstract

The wide use of antibiotics in multiple fields leads to their entry into the environment, challenging agriculture and ecology and potentially affecting maize seedling growth. In this study, maize variety Longken 10 was chosen as the experimental material. Subsequently, two antibiotics commonly utilized in production, namely oxytetracycline (OTC) belonging to the tetracycline class and ciprofloxacin (CIP) from the quinolone class, were selected. To comprehensively examine the impacts of these antibiotics on the phenotype, photosynthetic enzymes, nitrogen metabolism, and endogenous hormone contents of maize seedlings, a series of different concentration gradients (0, 3, 5, 30, 60, and 120 mg·L^−1^) were established, and the nutrient solution hydroponic method was employed. The results showed that, compared with the control group (CK), the activities of all indicators of maize seedlings were the strongest and the seedling growth was the most vigorous when the concentration of CIP was 5 mg·L^−1^ and that of OTC was 3 mg·L^−1^. The inhibitory effect of OTC on various indicators of maize seedlings was stronger than that of CIP. The underground parts of maize seedlings were more sensitive to OTC and CIP than the aboveground parts. Overall, maize seedlings exhibited a trend where high concentrations (30–120 mg·L^−1^) of antibiotics inhibited growth, while low concentrations (3–5 mg·L^−1^) promoted growth. The treatment groups with 3–5 mg·L^−1^ of OTC and CIP increased maize seedling growth phenotypes, the robust growth of seedlings with enhanced vitality, and the relative water content of maize leaves; decreased the relative electrical conductivity of maize leaves, indicating reduced cell permeability; increased the activities of leaf photosynthetic enzymes (PEPCase, RUBPCase, PPDK, NADP-ME, and NADP-MDH); increased the levels of hormones (IAA, GA, and ZR) in maize leaves and roots; decreased the levels of ABA and MeJA; increased the levels of nitrogen metabolism-related enzymes (GS, GOGAT, and GAD) in roots and leaves; decreased the GDH level; enhanced root activity and increased various root parameters (including average diameter, number of root tips, total volume, total root length, and root surface area), indicating vigorous root growth. Compared with CK, the treatment groups with 30–120 mg·L^−1^ of OTC and CIP reduced the phenotypes of maize seedlings, decreased the relative water content of maize leaves and increased the relative electrical conductivity of maize leaves, indicating enhanced cell permeability; reduced the activity of leaf photosynthetic enzymes, leading to weakened photosynthesis and decreased photosynthetic productivity; lowered the levels of IAA, GA, and ZR in leaves and roots of maize seedlings, and increased the levels of ABA and MeJA; decreased the levels of GS, GOGAT, and GAD in leaves and roots of maize seedlings, and increased the GDH level; reduced root activity, with the corresponding decrease in various root parameters.

## 1. Introduction

Maize, a major global staple crop, is vital for global food security as its yield and quality relate to food supply stability [1]. However, extensive antibiotic use in agricultural activities (e.g., livestock manure application and aquaculture wastewater irrigation) has led to prevalent antibiotic residues in soil and water, posing a threat to maize cultivation [1]. Antibiotics are applied in healthcare, livestock feed, and crop disease management [1]. In human, animal, and plant use, they are not fully metabolized, and pollution manifestations vary by field [1]. In the medical domain, China’s antibiotic consumption from 2016 to 2020 was 52–55% higher than that of EU countries like Germany and France [2,3,4]. Medical antibiotics are not fully absorbed by humans and can contribute to antimicrobial resistance (AMR) [5]. Unabsorbed portions are released or excreted into sewage, polluting urban water bodies and reaching agricultural soils [6,7]. In livestock production, veterinary antibiotics are the main source of antibiotic pollution in China, accounting for 70% of veterinary drug usage [8,9]. Research shows that 30–90% of them are excreted by animals into the environment, and antibiotic-containing waste used as fertilizer introduces them into the soil [8,9]. The long-term use of chemical pesticides has led to pest resistance, so antibiotic-based bactericides are increasingly used in agriculture [10]. During pest and disease control, these antibiotics spread and accumulate in the soil, intensifying contamination [10]. In China, the maize planting area is extensive, covering various soil types and climate conditions, from the Northeast Plain to the North China Plain and the Southwest region. We chose maize as the research object and the research results can largely represent the situation of crops affected by antibiotics under multiple different environmental conditions, which is more universally applicable and can provide a theoretical basis for addressing issues related to antibiotic pollution in maize planting in different production areas.

This study selected oxytetracycline (OTC), a tetracycline antibiotic with relatively high soil residue and a stable detection rate, and ciprofloxacin (CIP), a fluoroquinolone antibiotic, which are representative. The main types of tetracyclines (TCs) include tetracycline (TC), oxytetracycline (OTC), chlortetracycline (CTC), and their semi-synthetic derivatives [11]. In recent years, with the extensive application of tetracycline antibiotics, the residues of antibiotics in soil and water have attracted widespread attention. Studies have found that tetracycline antibiotics were detected in the surface soil, where poultry and livestock manure were applied, with the concentrations of oxytetracycline (OTC), tetracycline (TC), and chlortetracycline (CTC) being 0.350 mg·kg^−1^, 0.107 mg·kg^−1^, and 0.119 mg·kg^−1^, respectively [12]. Meanwhile, other studies have shown that the content of tetracycline antibiotics in vegetable base soil was 84.8 μg·kg^−1^, with the concentrations of oxytetracycline (OTC), tetracycline (TC), and chlortetracycline (CTC) being 9.6 μg·kg^−1^, 44.1 ug·kg^−1^, and 31.1 μg·kg^−1^, respectively [13]. It was found that the maximum detection concentrations of tetracycline (TC), oxytetracycline (OTC), and chlortetracycline (CTC) in the Yangtze River Basin were 11.16 ng·L^−1^, 18.98 ng·L^−1^, and 5.86 ng·L^−1^, respectively, with detection rates of 27.2%, 27.2%, and 7.1% [14]. The main types of fluoroquinolones (QNs) include norfloxacin (NOR), enrofloxacin (ENR), ciprofloxacin (CIP), lomefloxacin (LOM), and ofloxacin (OFX) [15]. Ciprofloxacin (CIP) was detected in a vegetable base, with concentrations of 19.76 μg·kg^−1^, 25 μg·kg^−1^, and 7.69 μg·kg^−1^, and the detection rates were all above 80% [16]. The content of fluoroquinolone antibiotics in a farmland soil was 0–195 μg·kg^−1^, with the concentrations of ciprofloxacin, enrofloxacin, and norfloxacin being 0–126.25 μg·kg^−1^, 0–99.4 μg·kg^−1^, and 0–1030 μg·kg^−1^, respectively [17]. It was found that the content of ciprofloxacin in soil samples was 0.27–0.40 mg·kg^−1^, and norfloxacin was 1.54–2.37 mg·kg^−1^ in sludge [18].

The combined risk quotient assessment shows that classic water systems like the Yellow River, Liao River, Hai River, Songhua River, and Yangtze River Basin are high-risk areas for antibiotics, with higher concentrations in water than in sediment [19]. In the Bohai Rim region, antibiotic content in rivers ranges from 0 to 353.13 ng·L^−1^, in a moderate ecological risk zone [20]. In China’s four major lake areas, antibiotic content varies with season and human flow, and high contents of TCs and SAs are typically found in water and sediment [20]. Sampling in 26 districts and counties of Harbin found the groundwater antibiotic concentration range was 0.02–612 ng·L^−1^ [21]. Eleven types of antibiotics (mainly sulfonamides and quinolones) were detected in the Wenyu River water [22]. High levels of quinolone antibiotics were found in Suzhou City’s water bodies [23]. Chiffre et al. found diclofenac, sulfamethoxazole, and carbamazepine had high pollution levels in a French rural catchment area, with concentrations from 566 to 2476 ng·L^−1^ [24]. In total, 54.2% of wastewater samples in Wisconsin, USA, contained antibiotics [25]. Vietnam’s surface water has higher detection concentrations of certain antibiotics compared to other countries [26]. In Curitiba, Brazil’s river water samples, amoxicillin was found at the highest detection concentrations, and the total antibiotic detection concentration range was 0.13–4.63 ng·L^−1^ [27]. In the Suquía River in Córdoba, Argentina, 15 types of antibiotics (mostly quinolones) were detected in sediment and surface water [28].

Antibiotics are spread through multiple pathways involving humans, animals, and microorganisms, and after being adsorbed by soil, they remain there, ultimately affecting the safety of crop production [29]. Currently, various types of antibiotics at different levels are frequently detected in different types of soil in China [30], and solutions to the problem of antibiotic residues in soil still need to be improved. In different functional soil types in Tianjin, the total concentration of antibiotics was measured to be (4.35–1.35) × 10^3^ μg·kg^−1^, with slight differences in antibiotic concentrations among different soil types. The amount of organic fertilizer used is an important influencing factor, and the content of TCs (1.34 × 10^3^ μg·kg^−1^) and QNs (0.1–36.25 μg·kg^−1^) is relatively high in dryland soil [31]. In the soil of vegetable fields in Chongqing, the content of tetracycline was 79.81 μg·kg^−1^, and the antibiotic content in vegetable fields near livestock farms was higher than that in others [32]. The extensive use of antibiotics in the livestock industry has significantly increased the risk of soil residues around it. In the upper reaches of the Minjiang River in Sichuan Province, where the livestock industry has been industrialized, tetracycline is the main type of antibiotic in the soil, and the sampling sites fall within the medium-risk category, causing adverse effects on the environment [33]. In the vegetable fields in the northern suburbs of Guangzhou, the concentration of tetracycline was measured to be 0–1036.52 μg·kg^−1^. Although the overall risk is low, attention should still be paid to the medium- and high-risk threats in some areas [34]. Antibiotic pollution and residue have become a global environmental concern. Research on soil samples collected in Al-Kharj, Saudi Arabia, found that the ecological risks of OT (34.52 μg·kg^−1^) and DC (26.54 μg·kg^−1^) were relatively high, while TC indicated a medium risk [35]. The residues of fluoroquinolone antibiotics in the soil of Switzerland and Australia range from 250 to 470 μg·kg^−1^ [36,37]. Residues of doxycycline and enrofloxacin were detected in agricultural soils in Selangor, Negeri Sembilan, and Malacca on the Malaysian Peninsula (36–378 μg·kg^−1^ DW) [38].

Oxytetracycline is a broad-spectrum rapid bacteriostatic agent that inhibits bacterial synthesis by quickly binding and occupying sites, thereby preventing the increase in peptide chains. When its concentration reaches a certain level, it can even have a bactericidal effect [39]. The impact of oxytetracycline on plants depends on the type of plant and its living environment. A large number of studies have been conducted based on hydroponic or soil culture experiments, adding different types of antibiotics to explore their effects on various plants. Based on research on aquatic plants, scenedesmus obliquus under oxytetracycline stress all showed stress effects, with a half-inhibitory concentration of 2 mg·L^−1^; concentrations of 10–20 mg·L^−1^ caused oxidative damage [40]. Previous studies on the toxic effects of oxytetracycline on ten economic crops found that its toxic response to root length was the best and could be used as a physiological indicator to evaluate its toxicity [41]. Based on research on terrestrial crops such as cereals and legumes, it was found that high concentrations of oxytetracycline significantly inhibited the germination of alfalfa seeds and had obvious inhibitory effects on root length, plant height, and chlorophyll synthesis [42]. Oxytetracycline inhibited the root development of alfalfa seedlings, and the root length and root surface area of wheat decreased with the increase in its dosage [43]. The degree of influence of oxytetracycline on the biomass of alfalfa and wheat depends on the level of addition; high concentrations of oxytetracycline showed inhibitory effects on both, but adding oxytetracycline under high phosphorus inhibition promoted growth [44]. Oxytetracycline first inhibited and then promoted the activity of antioxidant enzymes in maize seedlings, and the toxicity was reduced with the addition of zinc ions [45]. The inhibitory effect of oxytetracycline on the aboveground parts of rice was lower than that on the underground parts, and the underground parts had a stronger absorption capacity for oxytetracycline [46]. Oxytetracycline pollution at 30 mg·kg^−1^ significantly inhibited the biomass and underground growth and development indicators of rice [47]. Studies show that as oxytetracycline concentration rose, the physiological indicators of soybean and wheat seedlings decreased, yet wheat had a promoting effect under low concentrations, indicating different crop types respond differently to oxytetracycline [48,49]. Analyzing research data on vegetable crops, it was found that in hydroponic experiments, the inhibition rate of oxytetracycline on Chinese cabbage’s radicles and hypocotyls was significantly higher than in soil culture [50]. The inhibitory effect of oxytetracycline on Chinese cabbage was lower than that of chlortetracycline, and Chinese cabbage roots had higher resistance to both in lateritic red soil than in water solution [51]. Different vegetable varieties have different oxytetracycline accumulation levels related to vegetable root exudates, and the absorption rate is higher in water solution [52]. The accumulation of antibiotics in the roots of water celery was higher for enrofloxacin than for oxytetracycline; the accumulation of oxytetracycline in the roots of water celery was the largest [53]. Under oxytetracycline stress, the plant height, leaves, and yield of chili peppers all showed a downward trend [54]. At lower concentrations, oxytetracycline had no significant effect on the germination rate and germination index of Chinese cabbage, but had a significant inhibitory effect at high concentrations; the accumulation of oxytetracycline in the roots of pakchoi was higher than in the leaves [55]. Oxytetracycline had a significant inhibitory effect on the growth of tomato roots, and high concentrations of oxytetracycline inhibited the cell division of root cells [56].

Ciprofloxacin belongs to the quinolone (QNs) class, widely used since 1987 for infections caused by Gram-positive and Gram-negative bacteria, commonly treating severe infections in urinary, respiratory, and gastrointestinal tracts, and later extensively applied as a veterinary drug [57]. The extensive use of ciprofloxacin has led to its entry into soil through various means and subsequent absorption by plants, causing harm. Numerous scientific studies have discussed the hazards of ciprofloxacin in different plant fields, demonstrating the breadth of research. Studies have found that after ciprofloxacin stress, the chloroplast color of *Phaeodactylum tricornutum* fades at high concentrations, and the cell aggregation force increases, forming clusters; the photosynthetic system’s performance decreases over time; and the antioxidant system is inhibited [58]. The half-maximal inhibitory concentration for Navicula capitata is 3.07 mg·L^−1^, and its antioxidant system is damaged by ciprofloxacin stress but repaired through other system cycles and increased antioxidant enzyme activities [59]. The root iron plaque chelates ciprofloxacin, and a larger plaque means less plant root absorption and lower risk to the plant [60]. Another area of high research interest is the vegetable field. Ciprofloxacin can inhibit the germination of pakchoi seeds and has an inhibitory effect on the root elongation of pakchoi. Its exposure can increase the proportion of drug-resistant bacteria [61]. Lettuce is affected by ciprofloxacin from the seed germination stage to the growth of seedlings, and the yield shows a high inhibition and low promotion phenomenon. The application of ciprofloxacin can cause soil alkalization [62]. The inhibition rate of ciprofloxacin on root and bud lengths of pakchoi, maize, and radish is positively correlated with its concentration, with root elongation being the most sensitive, and pakchoi being the most sensitive among the three crops [63]. Regarding cereal crops, ciprofloxacin inhibits ryegrass’s germination potential and index, restricts root and bud length elongation (more on root length) [64]. It has no effect on wheat seed germination rate but inhibits root and bud length, and when combined with copper pollution, no obvious toxic effect is observed [65]. The concentration of ciprofloxacin is negatively correlated with the root activity of wheat; the wheat radicle is more sensitive to ciprofloxacin than the bud; the root of wheat has a higher inhibitory capacity for ciprofloxacin than the leaf; at high concentrations, it has a significant inhibitory effect on the antioxidant enzyme system and chlorophyll content of wheat [66]. Some studies also showed that ciprofloxacin has no effect on the germination rate of maize but causes severe oxidative damage to the maize root system, with relatively low oxidative damage to the bud [67].

Research on maize’s response to antibiotics is crucial for solving agricultural environmental problems and ensuring food security. Future efforts should integrate multiple disciplines (e.g., molecular biology, environmental science, microbiology) to reveal its molecular mechanism, develop stress-resistant varieties and remediation technologies, and optimize agricultural management with policy support. This study systematically examines the effects of two typical antibiotics on maize seedlings’ photosynthesis mechanism, nitrogen metabolism pathways, and endogenous hormone levels, clarifying the quantitative relationship between seedling growth and antibiotic levels. Longken 10 was selected as the material for the Hoagland nutrient solution hydroponic experiment. Typical antibiotics OTC and CIP were selected, and based on the previous preliminary experiments, the concentration gradients of 0, 3, 5, 30, 60, and 120 mg·L^−1^ were set to analyze and determine the phenotypes and physiological and biochemical indicators of the seedlings. This study can offer a reference for further understanding the ecological toxicity mechanism of antibiotics to field crops. Moreover, studying maize’s response mechanism to antibiotics is significant for alleviating pollution stress and ensuring its safe production.

## 2. Results

### 2.1. Phenotypes of Maize Seedlings

**Figure 1:**
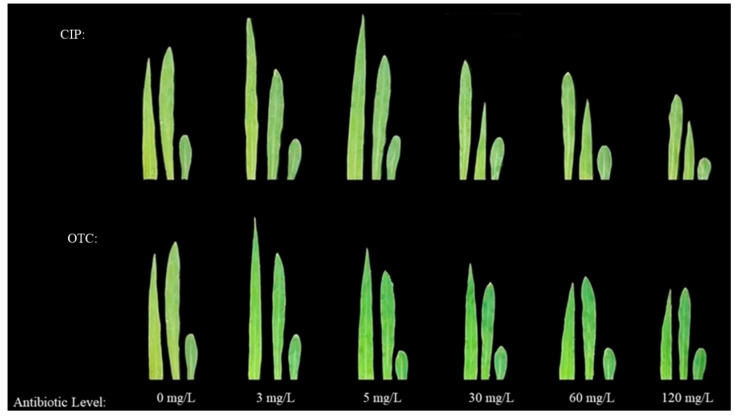
Effects of different concentrations of OTC and CIP on the morphological structure of maize seedling leaves.

**Figure 2:**
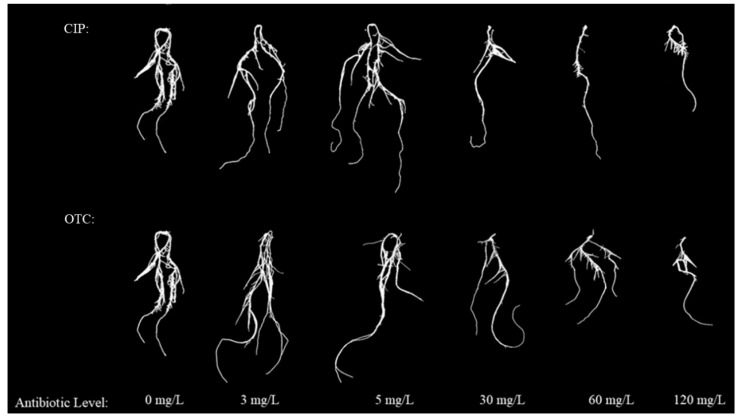
Effect of different concentration of CIP and OTC on maize seedling root structure.

The phenotypes of leaves and roots showed that, compared with CK, the fresh and dry weights of leaves and roots of maize treated with OTC first increased and then decreased. The promoting peak was reached at 3 mg·L^−1^, and the inhibitory effect began at 30–120 mg·L^−1^, reaching the inhibitory trough at 120 mg·L^−1^. After CIP treatment, compared with CK, the fresh and dry weights of leaves and roots of maize in each treatment first increased and then decreased. The promoting peak was reached at 5 mg·L^−1^, and the inhibitory trough was reached at 120 mg·L^−1^ (Figure 1 and Figure 2).

### 2.2. Relative Water Content of Maize Seedling Leaves

**Figure 3:**
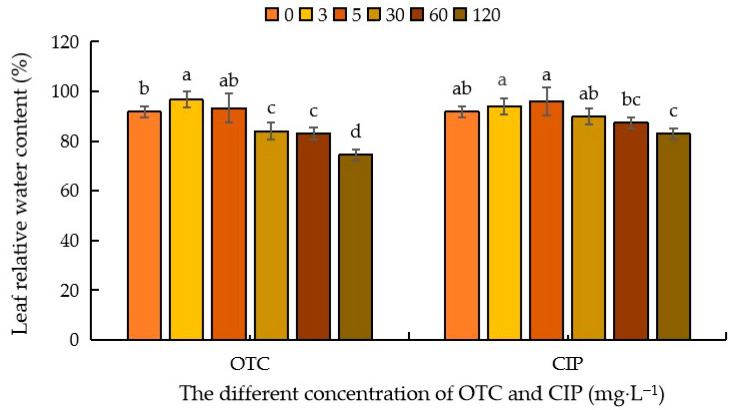
Effects of different concentrations of OTC and CIP on relative water content of maize leaves. Different letters within the same column indicate a significant difference at the 5% level.

As shown in Figure 3, compared with CK, under OTC treatment, the relative water content of maize leaves showed a trend of increasing first and then decreasing. When the OTC concentration was 3 mg·L^−1^, the relative water content of leaves showed an increasing trend, with a promotion rate of 5.24%; at 5 mg·L^−1^, it did not reach a significant difference level; at 30–120 mg·L^−1^, it began to show an inhibitory effect, with inhibition rates of 8.42%, 9.43%, and 18.89%, respectively. Compared with CK, after CIP treatment, there was an increasing trend at 3–5 mg·L^−1^, reaching a promotion peak at 5 mg·L^−1^, with promotion rates of 2.30% and 4.5%, respectively; at 120 mg·L^−1^, it reached an inhibition trough, with an inhibition rate of 9.62%; at 30–60 mg·L^−1^, it did not reach a significant difference level (Figure 3).

### 2.3. Relative Electrical Conductivity of Maize Seedling Leaves

**Figure 4:**
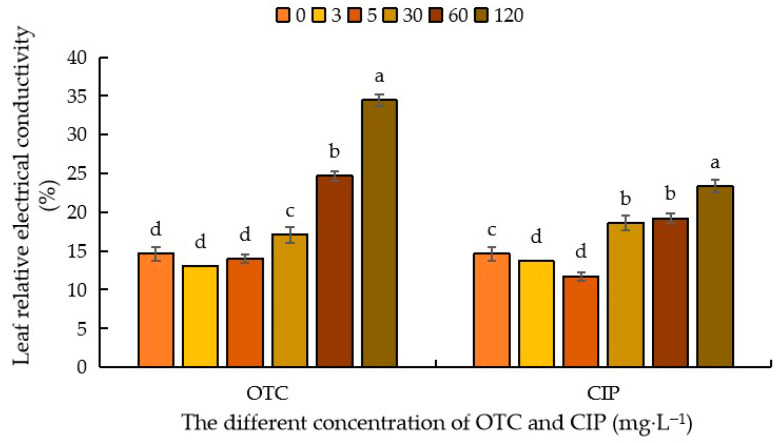
Effects of different concentrations of OTC and CIP on relative conductivity of maize leaves. Different letters within the same column indicate a significant difference at the 5% level.

Relative electrical conductivity is a key indicator for measuring cell membrane permeability and reflecting a plant’s ability to resist adverse external damage. As shown in Figure 4, compared with the control (CK), after OTC treatment, the relative electrical conductivity of maize leaves decreased when the concentration was 3–5 mg·L^−1^, with a decrease of 13.09% and 4.34%, respectively. As the OTC concentration increased, the relative electrical conductivity of the leaves increased by 16.65%, 68.45%, and 135.07%. After CIP treatment, compared with CK, when the antibiotic concentration was 3–5 mg·L^−1^, the relative electrical conductivity of the leaves decreased by 7.10% and 24.71%, respectively. As the antibiotic concentration increased, the relative electrical conductivity of the leaves gradually increased by 26.96%, 31.09%, and 59.48%. Comparing the two antibiotics, it can be seen that after OTC treatment, the relative electrical conductivity of the leaves was higher, indicating higher cell membrane permeability (Figure 4).

### 2.4. Root Activity of Maize Seedlings

**Figure 5:**
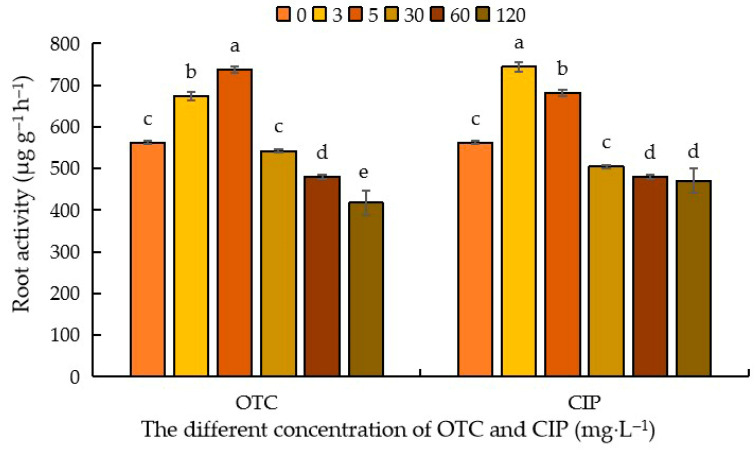
Effects of different concentrations of OTC and CIP on maize root activity. Different letters within the same column indicate a significant difference at the 5% level.

Root activity, in layman’s terms, refers to a composite indicator characterizing the absorption and biosynthetic capacity of roots. As illustrated in Figure 5, the impact of antibiotics on maize root activity exhibited an overall trend of initial enhancement followed by suppression, with variations observed based on antibiotic type and concentration. Following OTC treatment, compared to the control (CK), root activity demonstrated a stimulatory effect at 3–5 mg·L^−1^, with promotion rates of 31.27% and 19.99%, respectively. The peak stimulation occurred at 3 mg·L^−1^, reaching 737.46 μg g^−1^ h^−1^. Subsequently, an inhibitory effect emerged, with suppression rates of 3.56%, 14.46%, and 25.68% at 30–120 mg·L^−1^. The maximum inhibition was observed at 120 mg·L^−1^ (417.54 μg g^−1^ h^−1^). CIP treatment followed an analogous trend. Relative to CK, root activity was significantly enhanced at 3–5 mg·L^−1^ (34.86% and 47.22% promotion rates, respectively), peaking at 5 mg·L^−1^ (743.84 μg g^−1^ h^−1^). Inhibition then ensued, with suppression rates of 0.22%, 4.89%, and 6.97% at 30–120 mg·L^−1^, reaching its nadir at 120 mg·L^−1^ (470.05 μg g^−1^ h^−1^). These results indicate that elevated antibiotic concentrations exert inhibitory effects on root activity, with OTC demonstrating significantly stronger suppression than CIP (Figure 5).

### 2.5. Photosynthetic Key Enzyme Activities in Maize Seedling Leaves

The key enzymes of the C_4_ pathway in maize include PEPCase, RUBPCase, PPDK, NADP-ME, and NADP-MDH. PEPCase serves as the first potential selective inhibition site in C_4_ metabolism and is the initial critical enzyme in the C_4_ cycle. As shown in Table 1, PEPCase activity in maize leaves exhibited a biphasic response under both OTC and CIP treatments, characterized by initial stimulation followed by inhibition with increasing antibiotic concentrations. Under OTC treatment, PEPCase activity peaked at 3 mg·L^−1^ with a stimulation rate of 30.19%, then declined at higher concentrations. While activity remained above the control level at 5 mg·L^−1^, significant inhibition emerged at 30 mg·L^−1^ (7.99% suppression), intensifying to 19.47% at 60 mg·L^−1^ and reaching maximal inhibition (31.89%) at 120 mg·L^−1^. CIP treatment induced peak PEPCase activity at 5 mg·L^−1^ (25.30% stimulation), with maximal suppression (42.71%) occurring at 120 mg·L^−1^. NADP-ME represents the second potential selective inhibition site in C_4_ metabolism, catalyzing CO_2_ release into the Calvin cycle within bundle sheath cells. Studies demonstrate that NADP-ME (malic enzyme) activity followed a similar biphasic pattern, though peak stimulation concentrations differed between antibiotics: 3 mg·L^−1^ OTC (55.70% stimulation) versus 5 mg·L^−1^ CIP (55.32% stimulation), with no statistically significant difference. Both compounds reached maximal inhibition at 120 mg·L^−1^, with CIP exhibiting markedly stronger suppression (54.61%) compared to OTC (31.84%). PPDK, the third key site in the C_4_ pathway, functions as the rate-limiting enzyme for photosynthetic carbon assimilation and serves as the primary CO_2_ acceptor. Consequently, PPDK influences leaf respiration and starch synthesis. PPDK (pyruvate phosphate dikinase) activity under OTC treatment mirrored the aforementioned trend but with varying magnitudes: 55.70% and 43.70% enhancement at 3–5 mg·L^−1^ respectively, followed by progressive inhibition (2.68%, 32.01%, and 47.36% at 30–120 mg·L^−1^). In contrast, CIP treatment immediately suppressed PPDK activity (4.94% inhibition at 3 mg·L^−1^), indicating greater sensitivity to CIP. NADP-MDH, a malate decarboxylase localized in plant cytosol and chloroplasts, provides CO_2_ for the C_3_ cycle. Its activity under OTC treatment showed comparable dynamics: 26.18% and 11.58% enhancement at 3–5 mg·L^−1^, transitioning to inhibition (5.09%, 26.31%, and 33.40% at 30–120 mg·L^−1^). CIP treatment induced maximal NADP-MDH stimulation at 5 mg·L^−1^ (15.18%), but caused 11.51% inhibition at 3 mg·L^−1^. RUBPCase (ribulose-1,5-bisphosphate carboxylase) activity demonstrated positive correlation with photosynthetic rate, directly determining maize leaf photosynthetic capacity. OTC treatment maximally stimulated RUBPCase at 3 mg·L^−1^ (16.40% enhancement), with progressive decline culminating in 28.59% inhibition at 120 mg·L^−1^. CIP treatment produced peak stimulation at 5 mg·L^−1^ (21.86% enhancement) and maximal inhibition at 120 mg·L^−1^ (44.69%) (Table 1).

### 2.6. Hormones in Maize Seedling Root System

Plant endogenous hormones are trace substances synthesized internally that regulate physiological processes, primarily including auxins (IAA), cytokinins (ABA), gibberellins (GA), zeatin riboside (ZR), and jasmonates (MeJA). Methyl jasmonate (MeJA) exerts regulatory effects on the elongation of primary and lateral roots and is closely associated with plant damage response mechanisms. As shown in Table 2, compared to the control (CK), OTC treatment at 3–5 mg·L^−1^ exhibited inhibitory effects, with inhibition rates of 63.28% and 21.03%. The lowest activity occurred at 3 mg·L^−1^, while increasing antibiotic concentrations enhanced activity, demonstrating a synergistic effect at 30–120 mg·L^−1^ with promotion rates of 19.18%, 44.63%, and 51.45%. In contrast, CIP treatment reached its lowest activity at 5 mg·L^−1^ (inhibition rate: 39.27%), followed by increased MeJA activity at higher concentrations, showing synergistic effects at 30–60 mg·L^−1^ (promotion rates: 41.16%, 58.23%, and 66.29%). These results indicate that OTC exerts stronger inhibition on MeJA activity in maize roots than CIP. IAA, an auxin in plants, regulates root stem cell maintenance and adventitious root growth in maize. According to Table 2, OTC treatment induced a peak promotion rate of 67.71% at 3 mg·L^−1^ compared to CK, followed by 38.31% at 5 mg·L^−1^. However, inhibitory effects emerged at 30–120 mg·L^−1^, with rates of 24.25%, 36.08%, and 46.78%. CIP treatment initially elevated IAA activity at 3–5 mg·L^−1^ (promotion rates: 22.52% and 50.05%), peaking at 494.94 ng·g^−1^ (5 mg·L^−1^), but shifted to inhibition at 30–120 mg·L^−1^ (rates: 3.53%, 17.81%, and 41.28%). Thus, OTC exhibited significantly greater suppression of IAA than CIP. ABA, a cytokinin with broad biological functions, modulates root growth. Table 2 reveals dose-dependent ABA fluctuations under antibiotic treatments. OTC reduced ABA activity by 38.45% at 3 mg·L^−1^ (CK baseline), but inhibition diminished to 20.38% at 5 mg·L^−1^, transitioning to promotion (2.94–16.71%) at 30–120 mg·L^−1^. CIP initially suppressed ABA (minimum inhibition: 20.98% at 5 mg·L^−1^) before enhancing activity at 30–120 mg·L^−1^ (promotion rates: 5.39–27.92%). OTC demonstrated higher ABA inhibition than CIP. GA influences root growth gradually. Table 2 shows that higher antibiotic concentrations generally intensified GA suppression. OTC transiently promoted GA at 3–5 mg·L^−1^ (12.40% and 5.71%) but imposed inhibition (47.87–67.11%) at 30–120 mg·L^−1^. CIP initially elevated GA activity (19.78–23.73%, peak: 557.39 ng·g^−1^ at 5 mg·L^−1^) before suppressing it (9.11–22.88%) at higher concentrations. OTC’s inhibitory effect on GA surpassed CIP’s. ZR, a hormone involved in cell division and differentiation, displayed low-concentration promotion and high-concentration inhibition. OTC increased ZR activity by 21.85% and 13.76% at 3–5 mg·L^−1^ but suppressed it (39.06–59.43%) at 30–120 mg·L^−1^. CIP similarly enhanced ZR (18.74–39.83%, peak: 315.89 ng·g^−1^ at 5 mg·L^−1^) before inhibiting it (1.52–29.72%) at elevated doses. OTC’s ZR suppression was markedly stronger than CIP’s (Table 2).

### 2.7. Hormones in Maize Seedling Leaves

As shown in Table 3, following antibiotic treatment, the activity of methyl jasmonate (MeJA) in maize seedling leaves exhibited an initial suppression followed by promotion. Compared to the control (CK), oxytetracycline (OTC) treatment resulted in the lowest MeJA activity at 3 mg·L^−1^, with an inhibition rate of 36.06%. As the OTC concentration increased, MeJA activity gradually rose, showing an inhibition rate of 17.02% at 5 mg·L^−1^. At higher concentrations (30–120 mg·L^−1^), a stimulatory effect was observed, with promotion rates of 3.17%, 14.59%, and 35.34%, respectively. In contrast, ciprofloxacin (CIP) treatment led to a decline in MeJA activity at 3–5 mg·L^−1^, reaching its lowest level at 5 mg·L^−1^ (inhibition rate: 33.97%). Subsequent increases in CIP concentration (30–60 mg·L^−1^) elevated MeJA activity, with promotion rates of 4.68%, 11.71%, and 20.92%. These results indicate that OTC exerts a stronger inhibitory effect on leaf MeJA than CIP. Similarly, post-antibiotic treatment, indole-3-acetic acid (IAA) in maize leaves demonstrated concentration-dependent modulation: promotion at high concentrations and inhibition at low concentrations. OTC followed this trend but exhibited a distinct promotion peak at 3 mg·L^−1^ (46.24%), after which IAA activity declined progressively. At 30–120 mg·L^−1^, inhibitory effects were observed, with rates of 18.95%, 30.43%, and 34.06%. CIP treatment initially enhanced IAA activity at 3–5 mg·L^−1^ (promotion rates: 5.13% and 42.90%), peaking at 5 mg·L^−1^ (423.69 ng·g^−1^). Higher CIP concentrations (30–120 mg·L^−1^) suppressed IAA activity, with inhibition rates of 2.90%, 7.61%, and 16.58%. Notably, OTC’s inhibitory impact on leaf IAA was significantly greater than that of CIP. For abscisic acid (ABA), OTC treatment at 3–5 mg·L^−1^ reduced activity by 14.95% and 17.96% relative to CK, whereas rising antibiotic concentrations subsequently increased ABA levels, yielding promotion rates of 4.42%, 5.31%, and 22.92% at 30–120 mg·L^−1^. CIP induced an initial decline (inhibition rates: 10.85% and 17.97% at 3–5 mg·L^−1^, peaking at 203.50 ng·g^−1^) followed by activation (promotion rates: 6.07%, 15.17%, and 26.42%). OTC consistently exhibited stronger ABA suppression than CIP. Gibberellin (GA) activity under OTC treatment showed early promotion (13.94% and 10.08% at 3–5 mg·L^−1^) transitioning to inhibition (12.23%, 26.08%, and 42.04% at 30–120 mg·L^−1^). CIP initially elevated GA levels (10.40% and 15.38% at 3–5 mg·L^−1^, peaking at 517.20 ng·g^−1^), but higher concentrations suppressed activity (inhibition rates: 7.03%, 13.22%, and 25.71%). OTC’s GA-inhibitory effect surpassed CIP’s. Zeatin riboside (ZR) activity rose under OTC at 3–5 mg·L^−1^ (promotion rates: 10.93% and 6.88%) but declined sharply at 30–120 mg·L^−1^ (inhibition rates: 30.24%, 37.05%, and 41.94%). CIP similarly enhanced ZR at 3–5 mg·L^−1^ (6.88% and 10.93%, peaking at 181.99 ng·g^−1^) before suppressing it (9.02%, 14.15%, and 26.60%). OTC’s ZR inhibition was markedly stronger. Endogenous plant hormones, synthesized through metabolic processes, regulate physiological functions. Comparative analysis of maize seedling roots and leaves reveals that OTC imposes significantly greater hormonal suppression than CIP, with roots exhibiting higher sensitivity to both antibiotics than leaves (Table 3).

### 2.8. Nitrogen Metabolism-Related Enzyme Activities in Maize Seedling Roots

As shown in Table 4, the enzyme GAD in maize roots exhibited increased activity at low antibiotic concentrations and decreased activity at high concentrations. Compared to the control (CK), OTC treatment at 3 mg·L^−1^ resulted in peak GAD activity (68.27 μmol·min^−1^·g^−1^ FW), representing an 11.99% enhancement. As the OTC concentration increased, GAD activity gradually declined, showing inhibitory effects at 30–120 mg·L^−1^, with inhibition rates of 20.25%, 37.92%, and 59.03%, respectively. Similarly, CIP treatment induced peak GAD activity (69.42 μmol·min^−1^·g^−1^ FW) at 5 mg·L^−1^, corresponding to a 13.87% increase, followed by a gradual reduction at higher concentrations, with inhibition rates of 2.04%, 12.81%, and 21.94% at 30–120 mg·L^−1^. These results indicate that OTC exerts a stronger inhibitory effect on GAD activity than CIP. Glutamine synthetase (GS), a key enzyme in ammonia assimilation, significantly influences nitrogen transformation. Table 4 demonstrates that GS activity in maize roots initially increased and then decreased under antibiotic treatment, with OTC and CIP exhibiting similar overall trends but differing in concentration-dependent effects. Compared to CK, OTC treatment at 3 mg·L^−1^ achieved peak GS activity (0.3433 μ·g^−1^ FW·h^−1^), reflecting a 25.61% enhancement, while higher concentrations (30–120 mg·L^−1^) resulted in inhibition rates of 10.97%, 37.80%, and 48.78%. For CIP treatment, peak GS activity (0.3818 μ·g^−1^ FW·h^−1^) occurred at 5 mg·L^−1^, followed by inhibition rates of 7.32%, 34.15%, and 37.80% at 30–120 mg·L^−1^. Notably, OTC exhibited a more pronounced inhibitory effect on GS activity than CIP. Glutamate synthase (GOGAT), another critical enzyme in ammonia assimilation, displayed a similar biphasic response under antibiotic treatment (Table 4). OTC treatment at 3 mg·L^−1^ yielded peak GOGAT activity (18.54 μ·g^−1^ FW·h^−1^; 34.45% enhancement), whereas concentrations of 30–120 mg·L^−1^ induced inhibition rates of 28.33%, 36.91%, and 47.98%. CIP treatment at 5 mg·L^−1^ produced maximal GOGAT activity (21.91 μ·g^−1^ FW·h^−1^; 58.86% enhancement), with inhibition rates of 26.03%, 32.51%, and 47.62% at higher concentrations. These findings confirm that OTC exerts stronger suppression on GOGAT activity than CIP. In contrast to the preceding enzymes, glutamate dehydrogenase (GDH) activity in maize roots exhibited an inverse trend under antibiotic treatment (Table 4). OTC treatment at 3–5 mg·L^−1^ reduced GDH activity, with inhibition rates of 14.69% and 33.17%, reaching a minimum (48.10 μmol·min^−1^·g^−1^ FW) at 3 mg·L^−1^. However, GDH activity increased at 30–120 mg·L^−1^, showing enhancement rates of 2.99%, 12.04%, and 23.67%. Similarly, CIP treatment at 3–5 mg·L^−1^ suppressed GDH activity (inhibition rates: 33.95% and 19.87%), with a minimum (47.54 μmol·min^−1^·g^−1^ FW) at 5 mg·L^−1^, followed by activation at 30–120 mg·L^−1^ (enhancement rates: 6.52%, 16.75%, and 24.25%). Overall, OTC demonstrated greater inhibition of GDH activity than CIP (Table 4).

### 2.9. Nitrogen Metabolism-Related Enzyme Activities in Maize Seedling Leaves

As shown in Table 5, antibiotic treatment exhibited a biphasic effect on GAD enzyme activity in maize leaves, characterized by low-concentration stimulation and high-concentration inhibition. Compared with the control (CK), oxytetracycline (OTC) treatment induced peak GAD activity enhancement (34.54%) at 3 mg·L^−1^. Subsequent increases in OTC concentration progressively suppressed GAD activity, with inhibition rates of 9.42%, 27.45%, and 44.10% observed at 30–120 mg·L^−1^. For ciprofloxacin (CIP), maximal GAD stimulation (22.54% and 31.43%) occurred at 5 mg·L^−1^, followed by dose-dependent inhibition (6.00%, 14.72%, and 35.69% at 30–120 mg·L^−1^). These results indicate that OTC exerted stronger inhibitory effects on GAD activity than CIP. Similarly, glutamine synthetase (GS) activity demonstrated concentration-dependent modulation. OTC treatment achieved maximal stimulation (12.64%) at 3 mg·L^−1^, whereas inhibitory effects (13.97%, 21.98%, and 27.21%) emerged at higher concentrations (30–120 mg·L^−1^). CIP-induced GS activation peaked at 5 mg·L^−1^ (34.19%), with subsequent inhibition (0.81%, 13.89%, and 16.18%) at elevated concentrations. OTC consistently showed greater suppression of GS activity than CIP. The glutamate synthase (GOGAT) response followed an analogous pattern. OTC treatment elicited maximal activation (19.00%) at 3 mg·L^−1^, transitioning to inhibition (10.69%, 24.23%, and 44.13%) at 30–120 mg·L^−1^. CIP treatment induced peak stimulation (46.11%) at 5 mg·L^−1^, with identical inhibition rates (10.69%, 24.23%, and 44.13%) at higher concentrations. Comparative analysis confirmed OTC’s superior inhibitory potency on GOGAT activity. Contrastingly, glutamate dehydrogenase (GDH) exhibited inverse concentration–response relationships. OTC treatment suppressed GDH activity at 3–5 mg·L^−1^ (inhibition rates: 30.34% and 11.83%; minimum value: 61.84 μmol·min^−1^·g^−1^ FW at 3 mg·L^−1^), followed by activation (0.96%, 5.74%, and 15.54%) at 30–120 mg·L^−1^. CIP treatment similarly reduced GDH activity at 3–5 mg·L^−1^ (17.92% and 29.15% inhibition; minimum: 68.74 μmol·min^−1^·g^−1^ FW at 5 mg·L^−1^), with subsequent stimulation (0.32%, 7.22%, and 9.70%) at higher concentrations. OTC demonstrated stronger GDH inhibition than CIP (Table 5).

### 2.10. Parameters of Maize Seedling Root Systems

The root system serves as the primary organ for plants to absorb water and inorganic nutrients. As shown in Table 6, antibiotic-treated maize seedlings exhibited a biphasic response of stimulation at low concentrations and inhibition at high concentrations compared to the control (CK). The distinct peak stimulatory effects occurred at 3 mg·L^−1^ for oxytetracycline (OTC) and 5 mg·L^−1^ for ciprofloxacin (CIP). The average root diameter demonstrated concentration-dependent modulation: OTC at 3–5 mg·L^−1^ enhanced growth by 8.47% and 6.68%, whereas 30–120 mg·L^−1^ suppressed it by 38.44%, 50.56%, and 57.47%. CIP followed an analogous trend, with 3–5 mg·L^−1^ increasing diameter by 24.25% and 49.10%, while 30–120 mg·L^−1^ reduced it by 7.01%, 33.40%, and 50.88%. Root tip count under OTC treatment rose by 21.38% and 15.08% at 3–5 mg·L^−1^ but declined by 5.59%, 29.67%, and 47.75% at 30–120 mg·L^−1^. CIP induced 5.39% and 25.97% promotion at 3–5 mg·L^−1^, shifting to 56.64%, 62.04%, and 65.63% inhibition at 30–60 mg·L^−1^. Total root volume under OTC increased by 59.25%, 17.13%, and 1.03% at 3–30 mg·L^−1^ but decreased by 6.84% and 53.08% at 60–120 mg·L^−1^. CIP treatment yielded 25.69% and 40.07% enhancement at 3–5 mg·L^−1^, transitioning to 6.84%, 16.44%, and 41.09% suppression at higher concentrations. Total root length with OTC surged by 184.18% and 104.68% at 3–5 mg·L^−1^, then dropped by 15.52%, 30.28%, and 50.76% at elevated doses. CIP elicited 96.04% and 198.19% elongation at low concentrations, followed by 30.46%, 40.20%, and 58.94% reduction. Root surface area under OTC expanded by 161.68% and 76.91% at 3–5 mg·L^−1^ but contracted by 12.51%, 16.51%, and 34.77% at 30–120 mg·L^−1^. CIP treatment augmented surface area by 18.81% and 103.30% initially, then diminished it by 4.12%, 18.41%, and 41.38% (Table 6).

## 3. Discussion

Oxytetracycline is a tetracycline antibiotic that mainly exerts its effect by inhibiting bacterial protein synthesis. Ciprofloxacin is a fluoroquinolone antibiotic that interferes with DNA replication and transcription by acting on bacterial DNA gyrase and topoisomerase IV. Their different mechanisms of action imply that they may have distinct impacts on the soil microbial community around the roots of maize seedlings and on the physiological processes of the maize itself during seed germination and seedling growth. The experimental results indicate a slight variation in the phenotypes effects of two antibiotics on maize seedling growth. Following OTC treatment, the peak growth stimulation for both roots and leaves occurred at 5 mg·L^−1^, whereas CIP treatment induced peak stimulation at 3 mg·L^−1^. However, both antibiotics exhibited a general pattern of phenotypes promotion at lower concentrations (3–5 mg·L^−1^) and inhibition at higher concentrations (30–120 mg·L^−1^). These findings align with previous research on levofloxacin hydrochloride’s impact on maize seedling biomass, which demonstrated growth promotion at low concentrations (≤0.5 mg·L^−1^) and inhibition at high concentrations (≥5 mg·L^−1^) [68,69]. These observations are consistent with the former findings regarding sulfamethazine and ciprofloxacin’s stronger inhibitory effects on belowground versus aboveground growth in wheat [70]. The relative leaf water content (RLWC) serves as a critical indicator for assessing the water status of plant foliage [71]. Experimental results demonstrate that elevated concentrations (30–120 mg·L^−1^) of oxytetracycline (OTC) and peak concentrations (120 mg·L^−1^) of ciprofloxacin (CIP) significantly reduce RLWC below control levels. This phenomenon suggests a forced reduction in mesophyll cell turgor pressure, thereby inhibiting cellular expansion and ultimately impeding plant growth. Consequently, the equilibrium between foliar transpiration and root water uptake is disrupted [72,73]. Conversely, low antibiotic concentrations (3–5 mg·L^−1^) exhibit a marginal tendency to enhance RLWC, though both the promotion and inhibition rates remain below 20%, indicating negligible effects on leaf water status. The relative electrical conductivity reflects membrane permeability and serves as a quantitative measure of plant stress response. Under external stress, reactive oxygen species generated within plant tissues compromise cellular membranes, altering their permeability. Elevated relative electrical conductivity values correlate with increased membrane permeability and greater cellular damage [74,75]. Experimental data reveal that low antibiotic concentrations (3–5 mg·L^−1^) result in lower relative electrical conductivity compared to controls, indicating minimal membrane permeability and limited cellular damage. However, higher concentrations (30–120 mg·L^−1^) significantly elevate relative electrical conductivity, reflecting substantial membrane disruption. Notably, OTC demonstrates markedly greater membrane damage than CIP at high concentrations, underscoring the pronounced stress-induced injury caused by elevated antibiotic levels.

Under antibiotic stress, maize seedlings exhibit distinct variation trends in endogenous hormone levels between root systems and leaves. Plant growth and development are regulated by the interplay of various endogenous hormones, with abiotic stress or heavy metal contamination disrupting this balance and significantly elevating ABA levels. The interaction between antibiotics and the internal regulatory mechanisms of plants may be more complex than expected. Plants may activate compensatory or alternative regulatory pathways to maintain hormonal balance in response to the stress of antibiotics. Studies have revealed that cadmium stress increases ABA content while decreasing ZR content in soybean leaves, with an elevated ABA/ZR ratio restricting stomatal aperture and inhibiting root development [76]. Under low-temperature stress, maize shows increased ABA levels accompanied by reduced IAA and GA concentrations, suppressing seedling root growth [77]. Similarly, saline-alkaline stress leads to decreased IAA, GA, and ZR levels alongside elevated ABA content in maize [78]. The present experimental results demonstrate that high-concentration antibiotics (30–120 mg·L^−1^) significantly increase ABA and MeJA levels while reducing GA, IAA, and ZR concentrations. ABA enhances maize seedling stress tolerance through multiple mechanisms: regulating stomatal conductance to mitigate osmotic stress, boosting antioxidant capacity, and inhibiting root development [79]. Polyamines maintain plant cell vitality and delay senescence, whereas ABA exerts inhibitory effects by reducing cellular activity and suppressing growth in both leaves and roots. Elevated MeJA levels further exacerbate root growth inhibition, induce stomatal closure, and accelerate leaf tissue senescence, ultimately impairing crop development [80,81]. Optimal GA levels boost root cell division and growth, but high antibiotic concentrations suppress GA activity, harming root development. Since GA regulates the whole plant life cycle, its decline lessens the activity of key photosynthetic enzymes, inhibiting leaf photosynthesis [82,83]. Conversely, treatment with low-concentration antibiotics (3–5 mg·L^−1^) shows opposite trends: decreased ABA and MeJA levels alongside increased IAA, GA, and ZR concentrations in maize seedlings. The rise in MeJA, a defense signaling molecule, modulates stomatal transpiration, reducing water loss while enhancing leaf photosynthetic efficiency and mitigating antibiotic-induced stress [84]. Both IAA and ZR function as growth-promoting hormones, influencing cell elongation and differentiation [85]. Increased IAA stimulates lateral and adventitious root formation [86], while maintaining its balance with ZR enhances leaf anti-aging capacity and promotes overall crop vigor [87].

Experimental results demonstrate that antibiotic treatment exhibits a concentration-dependent effect on photosynthetic enzyme activity, with promotion at low concentrations (3–5 mg·L^−1^) and inhibition at elevated concentrations (30–120 mg·L^−1^). The peak promotion effects differ between OTC (observed at 5 mg·L^−1^) and CIP (achieving maximum stimulation at 3 mg·L^−1^). Under low-concentration antibiotic exposure, PEPCase, NADP-ME, PPDK, NADP-MDH, and RUBPCase activities showed increasing trends compared to controls. This stress response may correlate with mesophyll cell stomatal regulation, where stomatal closure restricts CO_2_ availability, triggering adaptive mechanisms to sustain photosynthetic carbon assimilation. Notably, PEPCase enhances photosynthetic efficiency in C_4_ plants by facilitating CO_2_ acquisition [88], while NADP-ME maintains equilibrium with other photosynthetic enzymes to confer stress tolerance [89]. At 30 mg·L^−1^ antibiotic concentration, photosynthetic enzyme activities exhibited overall inhibition relative to controls, indicating that high-level antibiotic contamination constrains photosynthetic performance through enzymatic regulation. High-concentration gentamicin impairs the photosynthetic electron transport chain in Scenedesmus obliquus, reduces chlorophyll a content, and disrupts energy balance, ultimately suppressing photosynthesis [90]. Similarly, sulfadiazine at elevated concentrations restricts growth and inhibits normal photosynthetic processes [91]. Furthermore, increasing antibiotic concentrations suppress PPDK activity, impair PEP formation, and diminish NADP-ME, NADP-MDH, and RUBP activities, thereby compromising CO_2_ generation and carbon assimilation, which detrimentally affects plant growth and development.

Crop physiological and biochemical metabolic pathways need multifaceted regulatory mechanisms. Specifically, amino acid synthesis/conversion and ammonium assimilation are regulated by key enzymes in nitrogen metabolism [92,93]. Maize’s physiological processes and economic yield are closely related to its nitrogen metabolism level, as key nitrogen metabolic enzymes notably affect final grain protein content [94]. Experimental results demonstrate that high concentrations of OTC and CIP (30–120 mg·L^−1^) reduce the activities of glutamine synthetase (GS), glutamate synthase (GOGAT), and glutamate decarboxylase (GAD) in maize roots and leaves, whereas low concentrations (3–5 mg·L^−1^) enhance these enzymatic activities. The level of GS activity profoundly impacts amino acid synthesis and nitrogen metabolism within plants. Elevated GS activity mitigates crop damage caused by excessive ammonium ions, whereas diminished activity exacerbates such effects [95,96,97]. GS participates in coupled reactions with GOGAT, constituting a critical component of amino acid biosynthesis and consequently influencing protein levels [98]. Furthermore, high concentrations of OTC and CIP (30–120 mg·L^−1^) increase glutamate dehydrogenase (GDH) activity in maize roots and leaves, while low concentrations (3–5 mg·L^−1^) suppress it, consistent with findings by Gulati et al. [99]. Under abiotic stress, GDH maintains physiological processes by facilitating NH_4_^+^ resynthesis and modulating nitrogen metabolism [100]. The impairment caused by high-concentration OTC and CIP stress disrupts nitrogen metabolic activities, which are governed by multiple key enzymes. The observed reduction in GDH activity reflects an adaptive response to preserve nitrogen homeostasis. Collectively, OTC treatment exhibits stronger inhibitory effects on nitrogen metabolism-related enzymes in maize roots and leaves compared to CIP, indicating greater phytotoxicity. Moreover, both OTC and CIP exert more pronounced inhibition in roots than in leaves, likely attributable to the specialized physiological functions of maize root systems.

The root system is a vital plant organ for absorbing water and inorganic salts, crucial for plant growth and development. Its growth status correlates with the aboveground parts. Under abiotic stress, roots dynamically adapt by adjusting their growth and vitality to maintain normal development. This study employed a hydroponic experiment as the foundational model, where roots first established contact with pollutants. Consequently, various root parameters were analyzed to evaluate the toxic effects of oxytetracycline (OTC) and ciprofloxacin (CIP) on maize seedlings. Compared to the control group (CK), antibiotic treatments demonstrated a dual-phase response in maize roots: low concentrations (3–5 mg·L^−1^) promoted growth, whereas high concentrations (30–120 mg·L^−1^) exhibited inhibitory effects. At 3 mg·L^−1^ OTC, maize seedlings reached peak stimulation in average diameter, root tip number, total volume, total root length, and root surface area, exhibiting vigorous root growth characterized by well-developed primary roots and increased fibrous roots. However, as OTC concentration increased, these parameters progressively declined, with a reduction in root tip count indicating diminished fibrous root formation. OTC stress impaired root functionality by reducing diameter, surface area, and volume, thereby compromising nutrient uptake and root anchoring capacity. CIP treatment followed a similar trend, with peak growth promotion observed at 5 mg·L^−1^. Higher CIP concentrations induced a significant negative correlation, leading to decreased root parameters. These findings align with prior research on tetracycline’s phytotoxic effects, where 10 μmol·L^−1^ tetracycline markedly suppressed rice root biomass, as well as studies documenting sulfamethazine and ciprofloxacin’s concentration-dependent inhibition of wheat root parameters [66,101].

The growth status, vitality, and metabolic activity of maize seedling roots can be characterized by root activity indicators. Experimental results demonstrate that antibiotic treatment exhibits a concentration-dependent effect on maize root activity, with low concentrations (3–5 mg·L^−1^) promoting growth and high concentrations (30–120 mg·L^−1^) exerting inhibitory effects. Notably, root reducing activity has been documented to increase under simulated abiotic stress conditions such as drought or saline-alkali environments [102]. This study reveals a negative correlation between root activity levels and increasing concentrations of oxytetracycline (OTC) and ciprofloxacin (CIP). As a quantitative indicator of physiological function performance in maize roots, the data show that treatments with 3 mg·L^−1^ OTC and 5 mg·L^−1^ CIP enhanced root activity by 31.27% and 47.22%, respectively, compared to the control group (CK). Future research should adopt interdisciplinary approaches (e.g., molecular biology, environmental science, microbiology) to elucidate the molecular mechanisms of maize response to antibiotics, develop stress-resistant cultivars and remediation technologies, and optimize agricultural management through policy interventions. Also, previous studies have also shown that under the growth conditions of soil, these antibiotics have different impact mechanisms on wheat [103]. Given that this experiment was conducted under Hoagland nutrient solution conditions, the results obtained may differ from those in actual field conditions. Therefore, future research should focus more on the study of antibiotics in actual soil environments, as the soil–plant system is highly complex and involves numerous biological and abiotic interactions. Antibiotics can interact with other soil components such as organic matter, clay minerals, and various chemical substances present in the soil. These interactions can alter the availability and mobility of the antibiotics themselves, as well as other essential nutrients required by plants. For instance, antibiotics may bind to soil organic matter, thereby changing the way plants obtain nutrients. Based on our experimental results in the Hoagland nutrient solution system, we found that at certain lower concentrations of the antibiotics under study, some changes occurred in plant growth parameters, which in some aspects could be regarded as potential stimulatory effects. However, these impacts are complex and often accompanied by changes in other physiological processes, such as alterations in hormone balance and root development. It should be noted that due to the aforementioned differences, directly extrapolating these findings to actual soil conditions is quite challenging. In natural environments, the combined influence of soil matrix, microbial communities, and other factors interacts with antibiotics and plants in ways that may alter the observed effects.

## 4. Materials and Methods

### 4.1. Experimental Materials

The maize variety “Longken 10” was procured from Longhui Maize Research Institute in Wuchang City, Heilongjiang Province. Oxytetracycline: Oxytetracycline hydrochloride soluble powder, veterinary drug approval number 120516200, was obtained from China Best Biological Co., Ltd. (Hefei City, Anhui Province, China), with physicochemical properties detailed in Table 7. Ciprofloxacin: Ciprofloxacin hydrochloride soluble powder, veterinary drug approval number 120152159, was sourced from China Zhonglong Shenli Co., Ltd (Hefei City, Anhui Province, China). The physicochemical characteristics of these two reagents are presented in Table 7.

### 4.2. Seedling Experiment

Before the experiment, the maize seeds were subjected to a disinfection process. We employed a common disinfection method, which involved soaking the seeds in a 10% (by volume) sodium hypochlorite solution for 15 min. Subsequently, the seeds were thoroughly rinsed with sterile distilled water at least three times to remove any residual sodium hypochlorite. The purpose of this disinfection process was to eliminate any potential contaminants, such as surface-attached microorganisms, which could interfere with the experiment and affect the accurate assessment of the impact of antibiotics on the seedlings.

(1) Seed Germination: we select the Longken 10 seeds and place them in a Petri dish (60 mm) for dark germination in a constant temperature incubator for 48 h.
(2) Transplanting: we select uniform and healthy seedlings without damage and transplant them into cultivation pots (60 × 30 × 15 cm), with 30 seedlings per pot. Add 10 L of nutrient solution to each pot. Set the light exposure to 12 h per day, with a temperature of 28 ± 1 °C. Change the nutrient solution every 3 days until the seedlings reach the two-leaf and one-heart stage, then proceed with antibiotic treatment.
(3) Antibiotic Treatment: we select uniformly grown seedlings and divide them into 11 treatment groups—Control group: 1/2 Hoagland nutrient solution, forming 1 control group; Treatment groups—Mix CIP and OTC at concentrations of 3, 5, 30, 60, and 120 mg·L^−1^ into 1/2 Hoagland nutrient solution, forming 10 treatment groups.
(4) Environmental Settings: The seedlings were cultivated in a growth chamber with controllable environmental parameters with a daytime temperature of 28 ± 1 °C and a nighttime temperature of 25 ± 1 °C. The light intensity was set at approximately 300 μmol·m^−2^·s^−1^. This intensity was chosen because it falls within the range typically used for growing maize seedlings and is suitable for supporting normal photosynthesis and growth activities. The light cycle was maintained at 12 h of light and 12 h of darkness. The relative humidity is maintained at 65–75%. The first day is marked as day 0. After continuous treatment for 4 days, samples of the parts to be measured are collected. Each index is repeated 3 times. These settings were carefully calibrated to simulate the natural day–night cycle and provide the seedlings with the optimal light environment to promote their growth and development.

### 4.3. Phenotypic Measurement of Seedling Growth

After 4 days of treatment, three uniformly developed seedlings were selected from each treatment group. The aerial parts were separated from the root systems using scissors, and their fresh weights were measured and averaged. Subsequently, the harvested aerial and root samples were individually placed in labeled kraft paper bags. The samples were then subjected to oven drying, with initial deactivation at 105 °C for 30 min followed by drying at 80 °C until a constant weight was achieved. Dry weights were measured using an electronic balance. The experiment was conducted with three biological replicates.

### 4.4. Determination of Leaf Relative Water Content

After 4 days of seedling treatment, three seedlings were selected from each treatment group. The first fully expanded leaf of each seedling was excised and weighed to obtain fresh weight (FW). The leaves were then immersed in distilled water (under dark conditions) for 24 h to achieve full turgidity before measuring saturated weight (SW). Subsequently, the samples were placed in kraft paper bags and oven-dried to constant weight to determine dry weight (DW). Leaf relative water content (RWC, %) was calculated using the formula: RWC = [(FW − DW)/(SW − DW)] × 100%.

### 4.5. Determination of Relative Electrical Conductivity of Leaves (EL)

On the 4th day after treatment of the seedlings, representative leaf samples were taken from each treatment. The fresh samples were rinsed clean with distilled water and cut into 0.5 g segments. They were then immersed in distilled water for 24 h. The electrical conductivity A was measured using a DDS-370A conductivity meter (Suzhou City, Jiangsu Province, China). The leaves were placed in a water bath and boiled in boiling water for 20 min, then cooled to room temperature, and the electrical conductivity B was measured. The relative electrical conductivity (%) = A/B × 100%.

### 4.6. Determination of Photosynthetic Enzyme Activities in Leaves

On the 4th day of maize seedling treatment, 0.5 g of leaf samples were collected and homogenized in a mortar with extraction buffer, followed by centrifugation (10 min, 15,000× *g*). The supernatant was collected for subsequent analysis. Enzymatic activities were measured using spectrophotometry at 450 nm after color development termination with the following commercial kits: RuBP carboxylase assay kit, plant PEPCase ELISA kit, plant PPDK detection kit, NADP-ME activity assay kit, and NADP-MDH detection kit (provided by Nanjing Jiancheng Bioengineering Institute for detection, http://www.njjcbio.com, Nanjing, China). All measurements were performed in triplicate.

### 4.7. Determination of Endogenous Hormone Activity

Samples were collected from the second fully expanded leaf and root system of maize seedlings on the 4th day after treatment. The samples were quickly frozen and ground in an ice bath with 2 mL of extraction solution until a uniform state was achieved. The ground samples were transferred to centrifuge tubes, shaken well, and refrigerated at 4 °C for 4 h. After centrifugation (8 min, 10,000× *g*), the supernatant was collected. The supernatant was passed through a column, dried to remove methanol, and diluted to a certain volume. The activities of IAA, ABA, GA, ZR, and MeJA in the leaves and roots of maize seedlings were determined by enzyme-linked immunosorbent assay [104]. The experiment was repeated three times, and the average value was calculated.

### 4.8. Determination of Nitrogen Metabolism-Related Enzyme Activities

On the 4th day post-treatment of maize seedlings, 0.5 g of root samples were collected and maintained on ice. The activities of nitrogen metabolism-related enzymes in roots were assayed using commercial kits for glutamate dehydrogenase (GDH), glutamine synthetase (GS), glutamate decarboxylase (GAD), and glutamate dehydrogenase (GDH) (provided by Nanjing Jiancheng Bioengineering Institute for detection, http://www.njjcbio.com, Nanjing, China). Absorbance values were measured at 20 s to 320 s intervals using a Shimadzu UV-2700i Ultraviolet-Visible Spectrophotometer (Beijing, China), with triplicate experimental replicates performed to obtain mean values.

### 4.9. Root System Parameters and Root Activity Measurement

On the 4th day after the treatment of maize, three seedlings were selected from each treatment. The roots were cleaned and scanned with the EPSON 1680 root scanner (Beijing, China). The root analysis (Winrhizo) was used to analyze various parameters of the maize root system, including average diameter, root tip number, total root volume, total root length and root surface area. On the 4th day after the treatment of maize, three seedlings with uniform growth were selected from each treatment to analyze their root activity. The TTC method was used for the determination [104].

### 4.10. Data Analysis

The data were expressed by the measured mean value, analyzed by SPSS19.0 (IBM SPSS Statistics, 2010), and compared by Duncan’s new complex difference method (α = 0.05), and origin 8 is used for drawing.

## 5. Conclusions

Oxytetracycline (OTC) and ciprofloxacin (CIP) show concentration-dependent biphasic effects on maize seedlings: low concentrations stimulate root parameters, and root activity; high concentrations inhibit them. Low concentrations enhance leaf relative water content (RWC), while high concentrations suppress it (OTC > CIP in inhibition); low concentrations increase leaf relative electrical conductivity (REC) and cell membrane permeability, inhibiting growth; low concentrations (3–5 mg·L^−1^) boost photosynthetic enzyme activity; high concentrations (30–120 mg·L^−1^) reduce it, lowering photosynthetic capacity and productivity. Low concentrations increase GS, GOGAT, and GAD activity but decrease GDH activity; high concentrations reverse this. OTC inhibits these enzymes more than CIP, with stronger suppression in belowground tissues. Low concentrations (3–5 mg·L^−1^) elevate IAA, GA, and ZR activity and reduce ABA and MeJA levels (promoting growth); high concentrations (30–120 mg·L^−1^) reverse this (inhibiting growth). OTC suppresses hormonal regulation more than CIP, and belowground tissues are more susceptible.

## Figures and Tables

**Table 1:** Effects of different concentrations of OTC and CIP on Photosynthetic enzyme activity in maize leaves.

Antibiotics	Concentrations (mg·L^−1^)	PEPCase (μmol·mg^−1^ pro·min^−1^)	NADP-ME (μmol·mg^−1^ pro·min^−1^)	PPDK (μmol·mg^−1^ pro·min^−1^)	NADP-MDH (μmol·mg^−1^ pro·min^−1^)	RUBPCase (μmol·mg^−1^ pro·min^−1^)
OTC	0	89.72 ± 1.37 ^c^	58.66 ± 0.55 ^c^	75.91 ± 4.19 ^c^	71.32 ± 0.35 ^c^	160.21 ± 0.44 ^ab^
	3	116.81 ± 3.91 ^a^	77.66 ± 5.21 ^a^	118.19 ± 5.77 ^a^	89.99 ± 1.11 ^a^	186.49 ± 6.07 ^a^
	5	94.02 ± 0.64 ^b^	64.97 ± 0.19 ^b^	109.08 ± 0.77 ^b^	79.58 ± 0.87 ^b^	167.38 ± 1.47 ^a^
	30	82.55 ± 0.63 ^d^	53.48 ± 2.67 ^d^	73.74 ± 1.40 ^c^	67.69 ± 2.16 ^d^	149.13 ± 4.57 ^ab^
	60	72.25 ± 2.15 ^e^	47.25 ± 2.30 ^e^	51.61 ± 0.97 ^d^	52.55 ± 1.25 ^e^	89.22 ± 63.92 ^d^
	120	61.11 ± 1.05 ^f^	39.98 ± 1.02 ^f^	39.96 ± 1.07 ^e^	47.43 ± 1.73 ^f^	114.41 ± 0.27 ^bc^
CIP	0	89.72 ± 1.37 ^c^	58.66 ± 0.55 ^c^	75.91 ± 4.19 ^b^	71.32 ± 0.35 ^b^	160.21 ± 0.44 ^c^
	3	98.17 ± 0.74 ^b^	68.33 ± 1.38 ^b^	72.16 ± 2.20 ^b^	63.11 ± 0.56 ^c^	169.98 ± 0.55 ^b^
	5	120.10 ± 3.25 ^a^	78.43 ± 5.11 ^a^	117.91 ± 8.42 ^a^	84.08 ± 3.66 ^a^	195.23 ± 5.99 ^a^
	30	77.18 ± 0.46 ^d^	55.49 ± 1.06 ^c^	60.67 ± 1.33 ^c^	52.16 ± 1.17 ^d^	136.40 ± 6.50 ^d^
	60	61.82 ± 1.65 ^e^	41.17 ± 0.67 ^d^	49.91 ± 0.98 ^d^	38.45 ± 0.78 ^e^	102.13 ± 2.12 ^e^
	120	51.40 ± 1.68 ^f^	26.62 ± 1.11 ^e^	26.97 ± 1.67 ^e^	31.09 ± 1.16 ^f^	88.62 ± 1.69 ^f^

Note: Data are expressed as the mean ± standard deviation. Different letters within the same column indicate a significant difference at the 5% level.

**Table 2:** Effects of different concentrations of OTC and CIP on endogenous hormones in roots.

Antibiotics	Concentrations (mg·L^−1^)	MeJA (ng·g^−1^)	ABA (ng·g^−1^)	GA (ng·g^−1^)	ZR (ng·g^−1^)	IAA (ng·g^−1^)
OTC	0	60.89 ± 1.99 ^d^	169.15 ± 1.60 ^c^	450.50 ± 5.63 ^c^	225.92 ± 4.35 ^c^	329.86 ± 8.14 ^c^
	3	22.36 ± 0.92 ^f^	104.12 ± 2.43 ^e^	506.34 ± 12.35 ^a^	275.28 ± 12.7 ^a^	553.19 ± 13.86 ^a^
	5	48.09 ± 1.63 ^e^	134.84 ± 7.37 ^d^	476.24 ± 4.29 ^b^	257.00 ± 8.89 ^b^	456.22 ± 17.57 ^b^
	30	72.57 ± 3.50 ^c^	174.12 ± 2.43 ^c^	234.86 ± 10.73 ^d^	137.68 ± 2.19 ^d^	249.86 ± 12.15 ^d^
	60	88.07 ± 1.22 ^b^	184.85 ± 1.30 ^b^	163.52 ± 11.37 ^e^	110.48 ± 2.17 ^e^	210.83 ± 11.20 ^e^
	120	92.22 ± 1.60 ^a^	197.42 ± 6.75 ^a^	148.16 ± 8.11 ^e^	91.66 ± 0.79 ^f^	175.56 ± 10.34 ^f^
CIP	0	60.89 ± 1.99 ^d^	169.15 ± 1.60 ^c^	450.50 ± 5.63 ^b^	225.92 ± 4.35 ^c^	329.86 ± 8.14 ^c^
	3	48.56 ± 1.21 ^e^	154.07 ± 4.63 ^d^	539.59 ± 9.50 ^a^	268.26 ± 4.96 ^b^	404.15 ± 12.41 ^b^
	5	36.98 ± 1.97 ^f^	133.66 ± 8.52 ^e^	557.39 ± 11.35 ^a^	315.89 ± 6.63 ^a^	494.94 ± 29.07 ^a^
	30	85.95 ± 2.26 ^c^	178.27 ± 2.24 ^bc^	409.48 ± 18.80 ^c^	222.49 ± 15.2 ^c^	318.21 ± 4.69 ^c^
	60	96.35 ± 3.61 ^b^	184.91 ± 4.70 ^b^	395.66 ± 7.61 ^c^	168.26 ± 4.96 ^d^	271.10 ± 3.76 ^d^
	120	101.26 ± 4.0 ^a^	216.38 ± 12.25 ^a^	347.43 ± 3.82 ^d^	158.77 ± 7.37 ^d^	193.71 ± 19.46 ^e^

Note: Data are expressed as the mean ± standard deviation. Different letters within the same column indicate a significant difference at the 5% level.

**Table 3:** Effects of different concentrations of OTC and CIP on hormone activity of maize leaves.

Antibiotics	Concentrations (mg·L^−1^)	MeJA (ng·g^−1^)	ABA (ng·g^−1^)	GA (ng·g^−1^)	ZR (ng·g^−1^)	IAA (ng·g^−1^)
OTC	0	71.36 ± 3.97 ^c^	248.07 ± 4.77 ^b^	448.28 ± 5.82 ^c^	167.37 ± 3.05 ^c^	296.50 ± 8.72 ^c^
	3	45.63 ± 2.07 ^e^	203.52 ± 9.62 ^c^	510.78 ± 6.89 ^a^	185.67 ± 1.96 ^a^	433.61 ± 11.72 ^a^
	5	59.22 ± 4.33 ^d^	210.99 ± 5.80 ^c^	493.46 ± 5.56 ^b^	178.89 ± 1.55 ^b^	394.03 ± 18.75 ^b^
	30	73.63 ± 3.66 ^c^	259.04 ± 2.04 ^b^	393.46 ± 5.56 ^d^	116.75 ± 1.31 ^d^	240.31 ± 13.53 ^d^
	60	81.77 ± 1.42 ^b^	261.25 ± 5.24 ^b^	331.38 ± 5.25 ^e^	105.36 ± 1.55 ^e^	206.27 ± 20.36 ^e^
	120	96.58 ± 0.48 ^a^	304.93 ± 14.29 ^a^	259.82 ± 3.03 ^f^	97.18 ± 2.92 ^f^	195.52 ± 1.78 ^e^
CIP	0	71.36 ± 3.97 ^c^	248.07 ± 4.77 ^c^	448.28 ± 5.82 ^c^	167.37 ± 3.05 ^c^	296.50 ± 8.72 ^b^
	3	57.24 ± 0.98 ^d^	221.15 ± 27.95 ^d^	494.91 ± 8.90 ^b^	181.99 ± 2.12 ^b^	311.72 ± 0.42 ^b^
	5	47.12 ± 0.40 ^e^	203.50 ± 2.98 ^d^	517.20 ± 9.42 ^a^	192.76 ± 8.32 ^a^	423.69 ± 19.91 ^a^
	30	74.70 ± 3.51 ^c^	263.12 ± 1.99 ^c^	416.78 ± 12.36 ^d^	152.27 ± 1.59 ^d^	290.91 ± 13.22 ^b^
	60	79.72 ± 1.54 ^b^	285.70 ± 3.79 ^b^	389.04 ± 11.83 ^e^	143.69 ± 1.88 ^e^	273.94 ± 1.96 ^c^
	120	86.29 ± 3.02 ^a^	313.61 ± 1.58 ^a^	333.02 ± 19.56 ^f^	122.85 ± 3.14 ^f^	247.34 ± 1.37 ^d^

Note: Data are expressed as the mean ± standard deviation. Different letters within the same column indicate a significant difference at the 5% level.

**Table 4:** Effects of different concentrations of OTC and CIP on enzymes related to root nitrogen metabolism.

Antibiotics	Concentrations (mg·L^−1^)	GAD (μmol·min^−1^·g^−1^ FW)	GS (μ·g^−1^ FW·h^−1^)	GOGAT (μ·g^−1^ FW·h^−1^)	GDH (μmol·min^−1^·g^−1^ FW)
OTC	0	60.96 ± 1.96 ^b^	0.273 ± 0.006 ^c^	13.79 ± 0.94 ^b^	71.98 ± 1.81 ^c^
	3	68.27 ± 0.81 ^a^	0.343 ± 0.029 ^a^	18.54 ± 0.98 ^a^	47.54 ± 1.24 ^e^
	5	65.43 ± 1.81 ^b^	0.287 ± 0.015 ^b^	14.88 ± 0.82 ^b^	57.68 ± 0.94 ^d^
	30	48.62 ± 2.22 ^c^	0.180 ± 0.010 ^d^	9.88 ± 0.16 ^c^	74.13 ± 3.37 ^c^
	60	37.85 ± 0.51 ^d^	0.170 ± 0.010 ^d^	8.80 ± 0.74 ^c^	80.65 ± 1.32 ^b^
	120	24.98 ± 0.13 ^e^	0.140 ± 0.010 ^e^	7.17 ± 0.16 ^d^	89.02 ± 1.18 ^a^
CIP	0	60.96 ± 1.96 ^b^	0.273 ± 0.006 ^c^	13.79 ± 0.94 ^c^	71.98 ± 1.81 ^d^
	3	63.77 ± 2.53 ^b^	0.333 ± 0.021 ^b^	18.62 ± 0.60 ^b^	61.40 ± 2.35 ^e^
	5	69.42 ± 2.19 ^a^	0.382 ± 0.007 ^a^	21.91 ± 0.81 ^a^	48.10 ± 1.58 ^f^
	30	59.72 ± 1.83 ^c^	0.253 ± 0.006 ^c^	10.20 ± 0.20 ^d^	76.67 ± 2.49 ^c^
	60	53.16 ± 1.18 ^d^	0.243 ± 0.023 ^d^	9.31 ± 0.88 ^d^	84.03 ± 1.20 ^b^
	120	47.59 ± 1.19 ^e^	0.170 ± 0.010 ^e^	7.22 ± 0.23 ^e^	89.44 ± 0.88 ^a^

Note: Data are expressed as the mean ± standard deviation. Different letters within the same column indicate a significant difference at the 5% level.

**Table 5:** Effects of different concentrations of OTC and CIP on enzymes related to nitrogen metabolism in maize leaves.

Antibiotics	Concentrations (mg·L^−1^)	GAD (μmol·min^−1^·g^−1^ FW)	GS (μ·g^−1^ FW·h^−1^)	GOGAT (μ·g^−1^ FW·h^−1^)	GDH (μmol·min^−1^·g^−1^ FW)
OTC	0	62.84 ± 1.77 ^c^	0.453 ± 0.006 ^c^	22.16 ± 0.69 ^bc^	88.78 ± 2.17 ^c^
	3	84.54 ± 0.76 ^a^	0.511 ± 0.033 ^a^	26.37 ± 1.76 ^a^	61.84 ± 1.47 ^e^
	5	72.77 ± 2.08 ^b^	0.474 ± 0.010 ^b^	23.88 ± 1.65 ^b^	78.28 ± 4.87 ^d^
	30	56.92 ± 0.87 ^d^	0.390 ± 0.006 ^d^	19.79 ± 1.07 ^d^	89.64 ± 2.79 ^c^
	60	45.59 ± 3.12 ^e^	0.354 ± 0.041 ^e^	16.79 ± 1.07 ^e^	93.88 ± 1.61 ^b^
	120	35.13 ± 3.65 ^f^	0.330 ± 0.026 ^e^	12.38 ± 0.74 ^f^	102.58 ± 3.14 ^a^
CIP	0	62.84 ± 1.77 ^c^	0.453 ± 0.006 ^c^	22.16 ± 0.69 ^c^	88.78 ± 2.17 ^b^
	3	77.00 ± 3.82 ^b^	0.554 ± 0.040 ^b^	26.75 ± 2.49 ^b^	75.29 ± 0.76 ^d^
	5	82.59 ± 2.35 ^a^	0.608 ± 0.014 ^a^	32.38 ± 0.74 ^a^	68.74 ± 4.15 ^c^
	30	59.06 ± 1.73 ^d^	0.450 ± 0.003 ^d^	20.47 ± 1.70 ^c^	89.06 ± 3.28 ^b^
	60	53.59 ± 1.28 ^e^	0.390 ± 0.003 ^e^	17.74 ± 0.41 ^d^	95.19 ± 1.85 a
	120	40.41 ± 0.98 ^f^	0.380 ± 0.015 ^e^	16.11 ± 0.47 ^d^	97.39 ± 2.75 ^a^

Note: Data are expressed as the mean ± standard deviation. Different letters within the same column indicate a significant difference at the 5% level.

**Table 6:** Effects of different concentrations of OTC and CIP on root parameters.

Antibiotics	Concentrations (mg·L^−1^)	Average Root Diameter (mm)	Number of Root Tips	Total Root Volume (cm^3^)	Number of Root Tips (cm)	Root Surface Area (cm^2^)
OTC	0	0.851 ± 0.105 ^a^	333.67 ± 7.37 ^c^	0.097 ± 0.004 ^b^	20.55 ± 1.63 ^c^	4.68 ± 0.37 ^c^
	3	0.923 ± 0.060 ^a^	405.00 ± 9.17 ^a^	0.155 ± 0.033 ^a^	58.41 ± 3.22 ^a^	12.25 ± 0.52 ^a^
	5	0.908 ± 0.077 ^a^	384.00 ± 8.00 ^b^	0.114 ± 0.009 ^b^	42.07 ± 4.29 ^b^	8.28 ± 1.04 ^b^
	30	0.524 ± 0.081 ^b^	315.00 ± 9.85 ^d^	0.098 ± 0.004 ^b^	17.36 ± 1.95 ^cd^	4.10 ± 0.15 ^c^
	60	0.421 ± 0.056 ^bc^	234.67 ± 5.51 ^e^	0.091 ± 0.006 ^b^	14.33 ± 2.13 ^de^	3.91 ± 0.16 ^cd^
	120	0.362 ± 0.052 ^c^	174.33 ± 9.29 ^f^	0.046 ± 0.006 ^c^	10.12 ± 0.89 ^e^	3.05 ± 0.06 ^d^
CIP	0	0.851 ± 0.105 ^bc^	333.67 ± 7.37 ^b^	0.097 ± 0.004 ^b^	20.55 ± 1.63 ^c^	4.68 ± 0.37 ^c^
	3	1.058 ± 0.050 ^ab^	351.67 ± 13.32 ^c^	0.122 ± 0.003 ^c^	40.29 ± 4.45 ^b^	5.56 ± 0.62 ^b^
	5	1.269 ± 0.394 ^a^	420.33 ± 17.01 ^a^	0.136 ± 0.006 ^a^	61.29 ± 1.23 ^a^	9.52 ± 0.50 ^a^
	30	0.792 ± 0.082 ^bc^	144.67 ± 6.66 ^d^	0.091 ± 0.004 ^d^	14.29 ± 1.01 ^d^	4.49 ± 0.21 ^cd^
	60	0.567 ± 0.048 ^cd^	126.67 ± 8.08 ^de^	0.081 ± 0.006 ^de^	12.29 ± 0.42 ^d^	3.82 ± 0.69 ^d^
	120	0.418 ± 0.081 ^d^	114.67 ± 9.29 ^e^	0.057 ± 0.004 ^e^	8.44 ± 0.17 ^e^	2.74 ± 0.08 ^e^

Note: Data are expressed as the mean ± standard deviation. Different letters within the same column indicate a significant difference at the 5% level.

**Table 7:** Structure and physicochemical properties of OTC and CIP.

Name	Formula	Molecular Weight (g·mol^−1^)	Structural Formula	CAS Number	Density (g/cm^3^)	Solubility (mg·L^−1^)	Log Kow
OTC	C_22_H_24_O_9_N_2_	460.434	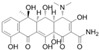	79-57-2	1.6340	H_2_O: slightly soluble	3.74
CIP	C_17_H_18_FN_3_O_3_	331.341	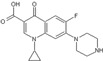	85721-33-1	1.461	H_2_O: almost insoluble	1.98

## Data Availability

The data presented in this study are available upon request from the corresponding author.
